# Improving macromolecular structure refinement with metal-coordination restraints

**DOI:** 10.1107/S2059798324011458

**Published:** 2024-12-03

**Authors:** Kaveh H. Babai, Fei Long, Martin Malý, Keitaro Yamashita, Garib N. Murshudov

**Affiliations:** a Institute of Molecular Biology and Biotechnology, Ministry of Science and Education, 11 Izzat Nabiyev, Baku, Azerbaijan; b MRC Laboratory of Molecular Biology Francis Crick Avenue CambridgeCB2 0QH United Kingdom; c Biological Sciences, Institute for Life Sciences, University of Southampton, SouthamptonSO17 1BJ, United Kingdom; d Structural Biology Division, Research Center for Advanced Science and Technology The University of Tokyo 4-6-1 Komaba, Meguro-ku Tokyo153-8904 Japan; Global Phasing Ltd, United Kingdom

**Keywords:** refinement, restraints, metal-coordination geometry, macromolecular crystallography, cryo-EM

## Abstract

Metals are essential components for the structure and function of many proteins. However, accurate modelling of their coordination environments remains a challenge due to the complexity and diversity of metal-coordination geometries. To address this, a method is presented for extracting and analysing coordination information, including bond lengths and angles, from the Crystallography Open Database. By using these data, comprehensive descriptions of metal-containing components are generated. A stereochemical information generator for a particular component within a specific macromolecule leverages an example PDB/mmCIF file containing the component to account for the actual surrounding environment. A matching process has been developed and implemented to align the derived metal structures with idealized coordinates from a coordination geometry library. Additionally, various strategies, depending on the quality of the matches, were employed to compile distance and angle statistics for the refinement of macromolecular structures. The developed methods were implemented in a new program, *MetalCoord*, that classifies and utilizes the metal-coordination geometry. The effectiveness of the developed algorithms was tested using metal-containing components from the PDB. As a result, metal-containing components from the *CCP*4 monomer library have been updated. The updated monomer dictionaries, in concert with the derived restraints, can be used in most structural biology computations, including macromolecular crystallography, single-particle cryo-EM and even molecular mechanics.

## Abstract 2

The Crystallography Open Database was mined to extract and organize metal environments. An algorithm and software were designed to apply the derived data to metal-containing components.

## 1. Introduction

The determination of the three-dimensional structures of macromolecules and their complexes with various ligand molecules is an important step in understanding the biological processes in which they participate. The most widely used experimental techniques for this purpose are macromolecular crystallography (MX) and single-particle analysis (SPA) using electron cryo-microscopy (cryo-EM). In both methods, particularly when data are limited to medium and low resolution, the experimental data alone are insufficient to precisely position all atoms. Therefore, Bayesian statistics, utilizing prior knowledge about the building blocks of macromolecules and ligand molecules, are employed. For this approach to be effective, accurate bond lengths, angles and torsion angles, along with their associated standard deviations, must be tabulated and stored in a monomer library (Vagin *et al.*, 2004 ▸). When new components are encountered, their stereochemical description should be created and provided to refinement and model-building programs. Such descriptions can be generated using high-quality software tools, including *eLBOW* (Moriarty *et al.*, 2009 ▸) from the *Phenix* package, *grade* (Smart *et al.*, 2021 ▸) from Global Phasing and *AceDRG* (Long *et al.*, 2017 ▸) from *CCP*4. Although these programs can generate stereochemical information for most chemical components, they encounter difficulties with metal-containing components. As a result, the model quality around the metal atoms in many macromolecular atomic structures is often lower than that of the rest of the atomic model.

The automatic definition of stereochemistry around metal atoms without additional information is a challenging problem. Bond lengths and angles depend on various factors, including the charge of the metal, its coordination geometry and the chemistry of the surrounding atoms. Additionally, the same metal can exist in two or more different states within the same components, depending on the protein environment. Another complication is that the bonding pattern around metal atoms in metal-containing components is often incomplete. Generally, it is not feasible to isolate a metal-containing component from its environment. Generating stereochemical information for such components in isolation and applying it later during refinement and model building is difficult, if not impossible. These components only become complete when they are within proteins and surrounded by protein and/or solvent atoms. Furthermore, in many cases metals are part of an active site, and during the catalytic reaction of macromolecules it is not uncommon for oxidation states, coordination geometry and stereochemical information to change (see, for example, Bolton *et al.*, 2024 ▸). In other words, the context is important. Recent statistical analyses of metal-binding sites in metalloproteins by Bazayeva *et al.* (2024 ▸) have also provided valuable insights into typical metal-coordination distances. These data serve as reference information for refinement and validation, highlighting the variability and complexity of metal interactions across different environments.

There have been several significant efforts to address the challenges of dealing with metals in macromolecular structures, most notably *checkMyMetal* and *metalPDB* (Zheng *et al.*, 2014 ▸; Putignano *et al.*, 2018 ▸). Additionally, there are software tools and data tables that focus on specific metals (Moriarty *et al.*, 2009 ▸; Touw *et al.*, 2016 ▸; Harding *et al.*, 2010 ▸). However, to effectively address the current issues in the PDB and to minimize future problems, it is essential to develop a sufficiently general and versatile tool that can handle most metal-containing components. Such a tool should be capable of generating accurate stereochemical information even when the metal is in a different environment.

This contribution describes a set of methods for extracting coordination information, along with corresponding bond lengths and angles, from the small-molecular database the Crystallography Open Database (COD; Gražulis *et al.*, 2009 ▸), and using these data to generate a comprehensive description of components, including details around the metal that account for the actual environment in which the metal atoms are situated. A method for generating context-dependent stereochemical information has also been developed and implemented.

## 2. Methods

### 2.1. Extraction and organization of the metal environment

#### 2.1.1. Selection of COD entries

Crystal structures from the Crystallography Open Database (COD; Gražulis *et al.*, 2009 ▸), determined using single-crystal X-ray diffraction with a resolution better than 0.82 Å and an *R* factor^1^ below 0.1, were selected for further analysis. While these criteria do not entirely eliminate incorrect structures, subsequent filtering steps ensure that the structures included in the statistical analysis are of adequate quality. Moreover, structures with partially occupied non-H atoms within the unit cell were omitted from the study. These selection criteria are similar to those employed by Long *et al.* (2017 ▸). From the filtered data, only entries containing at least one metal atom in the asymmetric unit were considered for further examination (Table 1 ▸).

#### 2.1.2. Generation of the metal environment

For each selected crystal structure, all atoms within three unit cells in each of the *x*, *y* and *z* directions were generated using all of the symmetry operators of the crystal. For each metal in the asymmetric unit, all atoms within the distance *d*_12_ ≤ α(*r*_1_ + *r*_2_) were extracted and saved in a file, where *d*_12_ is the distance between the considered atoms and *r*_1_ (metal) and *r*_2_ are their ‘covalent’ radii (Cordero *et al.*, 2008 ▸). Three sets of coordinates were generated with α = 1.1, α = 1.2 and α = 1.3. All files were then divided into two sets: (i) those without any metal–metal ‘bonds’ and (ii) those with at least one metal–metal ‘bond’. The total number of metal-environment structures generated was 429 579. Of these, 228 063 files, which did not contain any metal–metal bonds, were used for further analysis. It is important to note that a single crystal may contain multiple metals, either with the same identity or with different identities. Environments were extracted for all metal atoms within the asymmetric unit of the crystal.

The current coordination geometry classes do not include cases with metal–metal bonds. These will be considered in the future; however, in macromolecules, it is extremely rare to observe metal–metal interactions.

#### 2.1.3. Classification of metal environments

We began with 31 ideal metal-coordination classes (Table 2 ▸), denoted as ‘pre-existing’. To create coordination classes that are independent of the metal and ligand identity, the bond lengths between metal and nonmetal atoms were normalized to a value of 1. The limitations of this normalization are partially mitigated by employing full Procrustes matching with scaling (Dryden & Mardia, 2016 ▸; Appendix *C*[App appc]).

Metal-environment structures extracted from the COD were assigned to the coordination classes through an iterative process. Initially, all H atoms were removed from the files as a preprocessing step. For each file, we extracted all atoms with metal–nonmetal distances of less than 1.3 × (*r*_1_ + *r*_2_). Coordination class assignment was then attempted using combinatorial Procrustes analysis (Appendix *C*[App appc]). If the assignment was unsuccessful, atoms within a distance of 1.2 × (*r*_1_ + *r*_2_) were considered, followed by atoms within 1.1 × (*r*_1_ + *r*_2_). Upon successful class assignment, the metal and the corresponding atoms were extracted and saved as separate files.

This iterative method for assigning structures to coordination classes ensured that each structure was assigned to the class with the highest possible coordination number. This approach helped to minimize complications arising from slight variations in bond lengths that could affect the coordination geometry. For instance, a structure initially classified as octahedral could be reclassified as square planar if two opposite vertices are excluded due to slightly longer than expected bond lengths. Similarly, removing one vertex might shift the classification to square pyramidal.

Following the initial classification, the structures underwent additional review based on the following criteria.

(i) Structures with a Procrustes distance below 0.3 to any of the ‘ideal’ classes were considered to be properly classified. However, if the distance exceeded 0.2, hundreds of randomly selected structures were manually inspected.
(ii) Structures were reassessed if they exhibited an unusually low coordination number (≤4), even if their Procrustes distances were less than 0.2.
(iii) A review was conducted for structures where the number of members in the class was particularly small. The class was considered to be small if *N*_class_ ≤ min(30, 0.05*N*_metal_), where *N*_class_ is the number of members in a tentative class and *N*_metal_ is the number of cases with the considered metal and coordination number.
(iv) A random selection of structures was examined, regardless of their classification status.
(v) Within each coordination class, metal–nonmetal distances were calculated and any instances where these distances were outliers (*i.e.* if the distance was greater than *q*_3_ + 1.5 × IQR, where *q*_3_ represents the third quartile and IQR denotes the interquartile range) were subjected to additional scrutiny.
(vi) Additionally, hundreds of randomly selected structures from various classes were manually inspected to verify that the implemented methods were functioning as intended.

When structures could not be matched to any of the existing coordination classes, new idealized structures were created, and the matching and classification process was repeated. Consequently, the total number of coordination classes increased to 95 (Table 2 ▸). Table 3 ▸ lists the metal atoms with their likely coordination for the cases with more than 500 members. A table containing all elements with their classes can be found in the supporting information.

The above iterative process of assigning and defining classes allowed us to classify the majority, although not all, of the coordination environments within the data set (Table 1 ▸).

### 2.2. Metal-containing component description generation

The algorithm for generating stereochemical information involves three steps.

#### 2.2.1. Algorithm 1: metal–ligand description generation

(i) Algorithm 2 is executed to generate the initial stereochemical information along with a set of coordinates.
(ii) Algorithm 3 is applied to redefine the stereochemical information specifically around the metal atom(s).
(iii) The geometry is optimized using the current stereochemical information and the coordinates in the monomer CIF file are updated with the help of *Servalcat* (Yamashita *et al.*, 2021 ▸).

#### 2.2.2. Algorithm 2: initial metal–ligand description generation

(i) The monomer CIF file is read using *GEMMI* (Wojdyr, 2022 ▸) to extract the list of atoms and bonds.
(ii) The environment around the metal atoms is analysed and nominal charges are assigned to ensure that the charge on each metal atom does not exceed one of its most common oxidation states (Greenwood & Earnshaw, 1997 ▸). Generally, it is assumed that local nominal charges within a given environment should be made as close to zero as possible. It should be noted that the charges on ligand atoms are not necessarily zero, even locally. When the compound is inserted into the macromolecule, the metal atom may form additional bonds with other atoms, leading to alterations in nominal charges. The criterion of local minimality of charges ensures that metals do not have unreasonably large positive charges.At this stage, it is assumed that the bond orders for non­metal atoms are correctly assigned and that the monomer CIF file includes all H atoms. Adding missing H atoms is not attempted in this branch of the algorithm.
(iii) The metal atoms and all associated bonds are removed from the list. The information about the metal and its bonding is retained for later use.
(iv) Stereochemical information is generated using the standard *AceDRG* procedure (Long *et al.*, 2017 ▸), a conformer is generated using *RDKit* (Landrum, 2016 ▸) if needed and the conformer is optimized using *Servalcat* with the stereochemical parameters produced by *AceDRG*.
(v) The metal atoms and their bonding information are reintroduced. Tentative positions for the metal atoms are set based on the average positions of the atoms bonded to them, and tentative bond lengths are set to the sum of the ‘ideal’ covalent radii: *r*_1_ + *r*_2_.
(vi) The coordinates are optimized using *Servalcat*.

#### 2.2.3. Algorithm 3: update the stereochemical information around metal atoms

(i) The monomer CIF and an example model file (typically PDB or mmCIF) are read and all atoms bonded to the metals are extracted. At this stage ‘incorrect’ atoms are filtered out (Appendix *D*[App appd]).
(ii) The best-matching metal-coordination geometry class is extracted using Procrustes analysis (Appendix *C*[App appc]).
(iii) If an appropriate class is found, then the bond distances and angles are updated for each metal environment; otherwise, only bond lengths are updated. For this, metal–ligand distances extracted from the COD analysis are used. Bond lengths and angle statistics are calculated on the fly. For bond lengths, if necessary, multiple modes are detected (Appendix *A*[App appa]). For angle statistics calculations, the symmetrized von Mises distribution is used (Appendix *B*[App appb]).
(iv) The monomer CIF file is updated with the new bond lengths and angles, focusing only on the ligand information. All other bonds and angles are written to a JSON file for use by downstream programs. For updating, only the single most probable bond and angle information is written out. For the JSON file, multiple modes of distances are included. The JSON file also contains stereochemical information corresponding to all found coordination classes.
(v) The coordinates of the ligand are optimized using *Servalcat*.

### 2.3. Implementation

The algorithms for generating initial stereochemical information and coordinates for metal-containing components have been implemented in the *AceDRG* program. The matching, extraction, compilation and application of stereochemical information pertaining to metal environments have been incorporated into a new program, *MetalCoord*.

The *MetalCoord* program operates in two primary modes and one secondary mode.

(i) The *update* mode is utilized to update stereochemical information for metal-containing components available from the *CCP*4 monomer library. *MetalCoord* updates the information only around the metal atoms of the component. In this mode, the model file can be supplied by the user or automatically retrieved from the PDB, with the structure with the highest resolution being selected. This mode necessitates an active internet connection. If the provided model file contains multiple instances of the component, *MetalCoord* selects the one with the smallest *B* value and highest occupancy, disregarding the others.
(ii) The *stats* mode has been designed for the derivation of all stereochemical information for all instances of the component in the model file. In this mode, the program processes each instance of the component within the model file individually. Here, the model file must be supplied by the user.^2^
(iii) The *coord* mode provides basic information about coordination geometry classes indentified in the COD.

### 2.4. Program availability

*AceDRG* is available as part of the *CCP*4 suite, whereas *MetalCoord* can be accessed on GitHub at https://github.com/Lekaveh/MetalCoordAnalysis together with a tutorial describing its application. The program will also be included in the next version of *CCP*4. *Servalcat*, which now can perform geometry optimization and maximum-likelihood crystallo­graphic refinement, is available both from *CCP*4 and on GitHub at https://github.com/keitaroyam/servalcat. The entire monomer library, along with the updated entries, is available from an upcoming version of *CCP*4 as well as on GitHub at https://github.com/MonomerLibrary/monomers.git.

## 3. Results and discussion

Our primary objective was to update the descriptions of metal-containing components provided by *CCP*4 (Agirre *et al.*, 2023 ▸), as they have not been revised since their introduction in the early 2000s (Vagin *et al.*, 2004 ▸). Although some frequently used components, such as haem, vitamin B_12_ (monomer codes HEM and B12) and certain iron–sulfur clusters (for example monomer codes SF4, SF3 and FS2), have been sporadically revised and manually corrected, there have been no systematic efforts to review and amend all metal-containing components. This revision is long overdue, and we are now addressing these issues.

To update the descriptions, we initially reviewed all 756 entries (as of February 2024) in the Chemical Component Dictionary (CCD; Dimitropoulos *et al.*, 2006 ▸) that contain metal atoms. While many of these entries are correct, we manually assessed each one to identify and rectify potential chemical inaccuracies. We discovered that at least 50 of the CCD entries exhibit varying degrees of inaccuracy. Some issues relate to structural integrity, while others could lead to incorrect chemical interpretations. It is important to note that in many cases the structures in the PDB entries are correct; however, their chemistry from the CCD does not meet the same standards. This discrepancy is presumably due to miscommunication between the PDB and depositors.

Problematic cases could be roughly divided into two classes.

**Figure 1:**
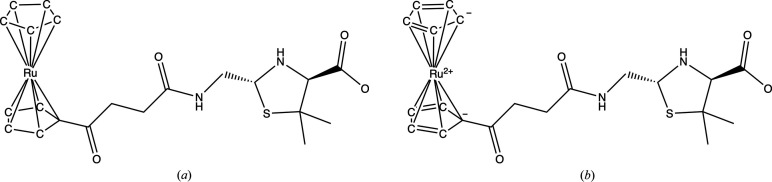
An example illustrating the importance of correct bond orders for structure interpretation is one of the sandwich structures present in the PDB. Both cyclopentadienyl rings must be planar with a nominal charge of −1. All C atoms on these rings must be *sp*^2^-hybridized. (*a*) From the CCD, the bond orders on both relevant rings are single, with no charge. Most programs interpreting this molecule with these bond orders will assume that all C atoms are *sp*^3^-hybridized, with two H atoms attached. Moreover, the metal atom (Ru) will be assumed to be neutral. (*b*) The bond orders and nominal charges on the cyclopentadienyl rings have been corrected. Now the rings are aromatic with a nominal charge of −1, and both rings are planar. According to *MetalCoord*, the coordination class is sandwich_5_5. Note that the structure, for example, in PDB entry 4xxr appears to be structurally sound (Lewandowski *et al.*, 2015 ▸). These figures were produced using *ChemDraw* version 23.01.

**Figure 2:**
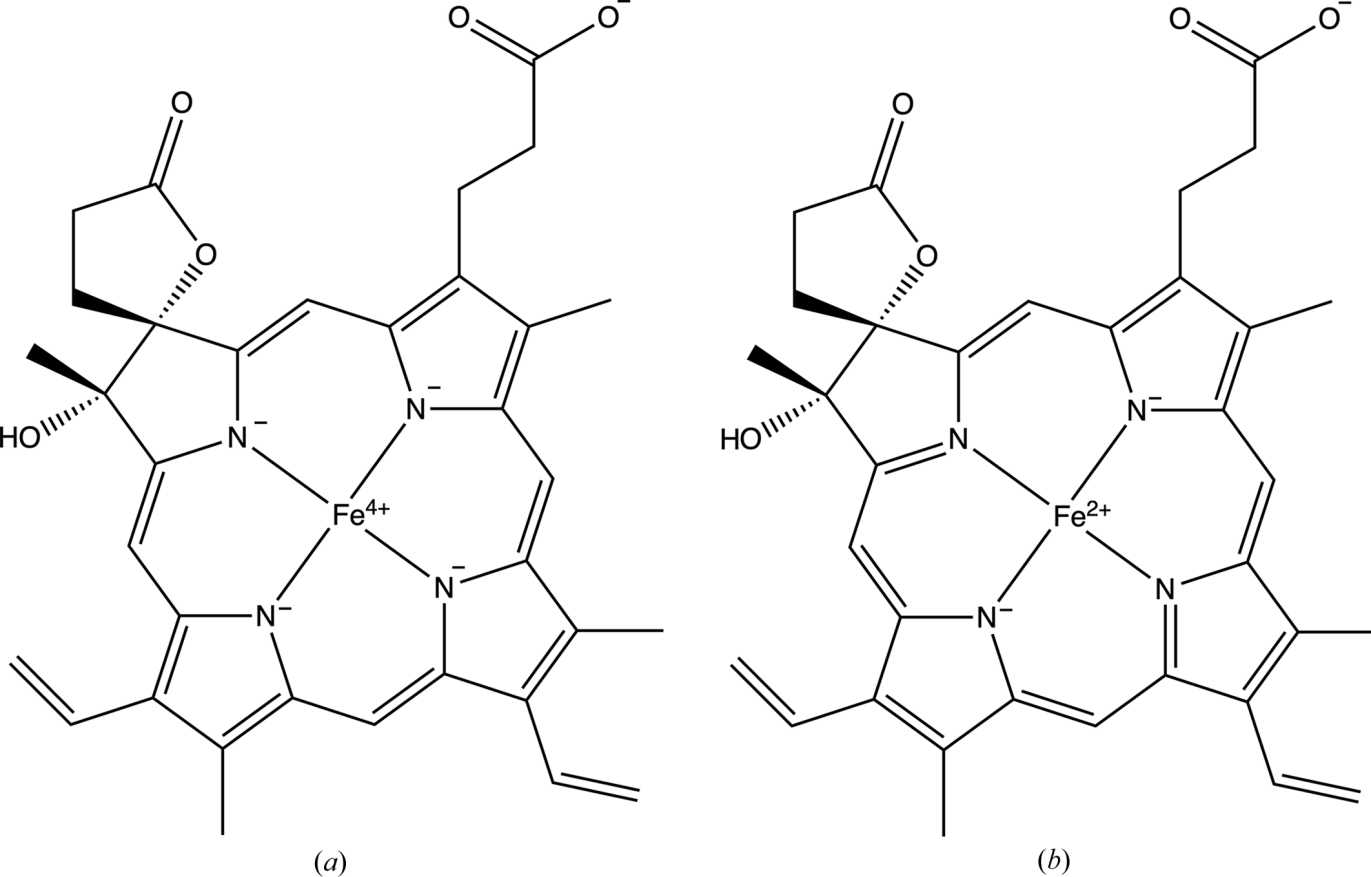
An example illustrating the importance of bond orders for the chemical interpretation of compounds within a macromolecule is shown. (*a*) In HDD from the CCD (as of February 2024), all N atoms have two single bonds within the rings, meaning they each can carry a −1 charge. This would result in the Fe atom having a +4 charge. However, within the protein, the iron of the haem often interacts with one or two amino-acid residues that can accept one or two more electrons. In other words, within the protein, the charge of the metal atom can increase. This would imply that the iron could have more than a +4 charge, which is not very likely. (*b*) After correcting the bond orders, the Fe atom now has a nominal charge of +2, and within the protein it can have a +3 charge. Note that both structures could exist as resonance forms. However, the structure in (*a*) would have higher energy compared with the structure in (*b*). When representing a structure, it seems reasonable to select the most probable one. The main difference between the structures in (*a*) and (*b*) is that the structure in (*a*) has 14 double bonds, while the structure in (*b*) has 13. The two extra electrons in structure (*a*) come from the Fe atom, making it +4. These figures were produced by *ChemDraw* version 23.01.

(i) Incorrect bond orders and missing H atoms were prevalent issues. Nearly all sandwich-like structures exhibited similar problems.^3^ In these cases, each ring must nominally carry a charge of −1, which implies that the metal attached to the ring must have a positive charge no less than the number of such rings. The ring must contain two double bonds, and all C atoms must be in an *sp*^2^-hybridization state. If the chemistry of the input is incorrect, stereochemical information-generating programs will be unable to produce chemically reasonable structures. Fig. 1 ▸ illustrates one such case, CCD entry JSD.
(ii) Errors affecting chemical interpretation: In several instances, particularly in haem-like structures,^4^ although the structures are correct, the chemical interpretation may be incorrect. For example, in HDD (Fig. 2 ▸) all N atoms of the pyrrole (or similar) groups have single bonds. This suggests that each must take one electron from the Fe atom, resulting in Fe^4+^. Given that the oxidation state of the metal can only increase within the protein, iron would end up with a higher positive charge than +4. This does not seem to be plausible. Correcting the bond orders allows easier interpretation; for instance, iron is +2 within the haem, and when the tyrosine of the molecule catalase attaches to it, iron will have a +3 charge. Fig. 2 ▸ illustrates this situation.

Out of 884 CCD metal-containing entries, we updated 809 using *AceDRG*, *MetalCoord* and *Servalcat*. This includes all non-obsolete metal-containing ligands containing more than one atom. We also excluded eight ligands containing boron clusters. Before updating, we needed to correct the chemistry of over 90 of them. Besides employing the update monomer library, it is generally recommended to use *MetalCoord* in *stats* mode to generate external restraints prior to macromolecular structure refinement. This ensures that the correct coordination geometry is identified and that the corresponding bond lengths and angles are applied.

## 4. Examples of application

From refinements of numerous structures while testing the updated *CCP*4 monomer library, we present a few example cases to demonstrate the improvement in refinement stability and structure model quality.

The structures shown in this section were re-refined using *Servalcat* employing the updated monomer library and restraints based on *MetalCoord* analysis. The cryo-EM SPA structure refinements were carried out in the *refine_spa_norefmac* mode and the crystal structure refinements in the *refine_xtal_norefmac* mode against structure-factor amplitudes. The refined structures, along with the scripts used, are publicly available at https://doi.org/10.5281/zenodo.13694559. The refinement statistics are briefly reported in Supplementary Table S1 and selected external restraints used during refinement are listed in Supplementary Table S2.

### 4.1. Haem-like components

Haem-like cofactors which bind a metal cation in their centre play fundamental roles in numerous large biomolecular complexes, including photosystems and respiratory complexes.

**Figure 3:**
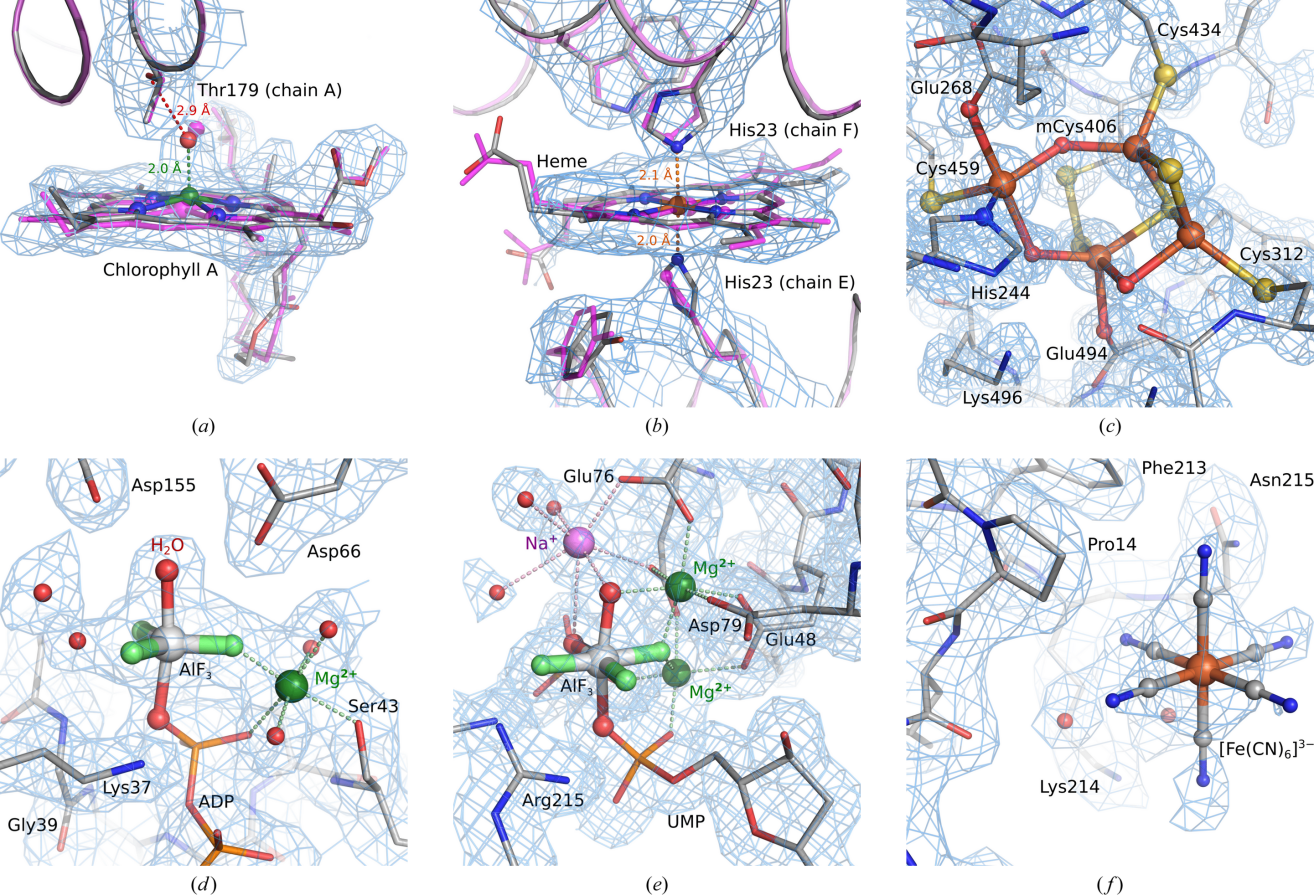
Structures refined in *Servalcat* using the updated monomer library and restraints from *MetalCoord*. The metal-containing compounds are highlighted in ball-and-stick representation. Water molecules are shown as red spheres. Coordination of other surrounding metal atoms and selected interactions are depicted as dashed lines. Atoms are coloured by their element: carbon, grey; nitrogen, blue; oxygen, red; sulfur, yellow; phosphorus, light orange; iron, dark orange; fluorine, light green; magnesium, dark green; sodium, purple. The figures were prepared using *PyMOL* 3.0 (Schrödinger). The refinement statistics are reported in Supplementary Table S1 and the relevant restraints are listed in Supplementary Table S2. (*a*, *b*) The cryo-EM SPA structure of monomeric photosystem II from *Synechocystis* (PDB entry 6wj6; Gisriel *et al.*, 2020 ▸). The originally deposited structure model is shown in magenta. The new monomer library in concert with refinement by *Servalcat* improved the modelling of the coordination of the magnesium cation in chlorophyll A (monomer code CLA) (*a*) as well as the coordination of the iron cation in haem (monomer code HEM) (*b*). The density was resampled (rate 1.5) and sharpened in *Coot* (Emsley *et al.*, 2010 ▸). (*c*) The hybrid iron–sulfur–oxygen cluster (monomer code FS2) in the hybrid cluster protein crystal structure (PDB entry 1w9m; Aragão *et al.*, 2008 ▸). mCys denotes *S*-mercaptocysteine. The 2*mF*_o_ − *DF*_c_ density map is contoured at a 2σ level. (*d*) Trigonal bipyramidal aluminium coordination in aluminium trifluoride (monomer code AF3) in the crystal structure of nitrogenase-like dark-operative protochlorophyllide oxidoreductase complex, chain *A* (PDB entry 2ynm; Moser *et al.*, 2013 ▸). The 2*mF*_o_ − *DF*_c_ density map is contoured at a 1σ level. (*e*) Octahedral aluminium coordination in aluminium trifluoride (monomer code AF3) in the crystal structure of dUTPase (PDB entry 4dl8; Hemsworth *et al.*, 2013 ▸). The 2*mF*_o_ − *DF*_c_ density map is contoured at a 1σ level. (*f*) Ferricyanide [Fe(CN)_6_]^3−^ (monomer code FC6) modelled with a partial occupancy of 0.8 in the crystal structure of bilirubin oxidase, chain *A* (PDB entry 6i3j; Koval’, Švecová *et al.*, 2019 ▸). The 2*mF*_o_ − *DF*_c_ density map is contoured at a 0.9σ level.

The beneficial impact of the updated library can be shown on the structure of monomeric photosystem II from *Synechocystis* (PDB entry 6wj6) determined using cryo-EM SPA at a resolution of 2.58 Å (Gisriel *et al.*, 2020 ▸). Our re-refinement improved the chemical correctness of the model as well as its agreement with the experimental density. The updated dictionary for chlorophyll A (monomer code CLA) allowed modelling of the magnesium cation out of the porphyrin plane (Fig. 3 ▸*a*). This enabled interaction with Thr179 via a water molecule. It should be noted that the maximum coordination number of the magnesium ion in chlorophyll A was set to five for the generation of the restraints for refinement (option -c 5 for *MetalCoord* in *stats* mode). Otherwise, irrelevant C atoms close to some magnesium ions were taken into account due to inaccurate input coordinates, which causes an incorrect increase in the magnesium coordination number to six. When using the further refined coordinates, *MetalCoord* interpreted these problematic cases correctly (*i.e.* a maximum coordination number of five) without any extra option being specified. Furthermore, in this structure model, the modelling of the iron-cation coordination in the haem molecules (monomer code HEM) with the neighbour histidine residues was considerably improved (Fig. 3 ▸*b*).

### 4.2. Hybrid iron–sulfur–oxygen cluster

The dictionary for the hybrid iron–sulfur–oxygen cluster (monomer code FS2) was incorrectly defined in the *CCP*4 monomer library in the past. The outlier analysis in *Servalcat* of the crystal structure of the hybrid cluster protein (PDB entry 1w9m), solved at a resolution of 1.35 Å (Aragão *et al.*, 2008 ▸; Fig. 3 ▸*c*) using the old dictionary (from *CCP*4 version 9.0.004), reported 14 bond-length and 16 bond-angle outliers with a *Z*-score higher than 5 for atoms of the FS2 monomer, despite the structure being correct. Consequently, this dictionary was manually revised (with FE5—FE6, FE6—FE7, FE5—O1 and FE8—O9 bonds removed, as they were either redundant or incorrect) and subsequently optimized in *MetalCoord*. Refinement using the updated dictionary resulted in only one significant outlier: a distance between the FE7 atom of the cluster and the hydroxyl group of Glu268. Such specific molecular interactions cannot be adequately described in a component dictionary file. Nevertheless, *MetalCoord* provides an analysis that generates external restraints suitable for a particular structure when an input model file is provided (see Appendix *D*1). In this case, the restraints generated for the cluster also include the ‘ideal’ value for the problematic distance mentioned (1.99 ± 0.13 Å), corresponding to trigonal bipyramidal coordination geometry, which is close to the distance observed in the deposited structure (2.14 Å).

Although the monomer library can be considered to be a reasonable starting point for metal-containing components, we also recommend running *MetalCoord* in *stats* mode while specifying a structure in the input. This will provide additional restraints suitable for the particular case, including molecular interactions.

### 4.3. Aluminium coordination depending on chemical context

The exact conformation of a molecule generally depends on its chemical environment. In the case of metal-containing components, the surrounding environment can also influence the metal-coordination geometry. For instance, the Al atom in aluminium trifluoride (monomer code AF3) within the nitrogenase-like dark-operative protochlorophyllide oxido­reductase complex (PDB entry 2ynm; Moser *et al.*, 2013 ▸) exhibits trigonal bipyramidal coordination (Fig. 3 ▸*d*), whereas it adopts an octahedral (square bipyramidal) coordination (Fig. 3 ▸*e*) in the dUTPase (PDB entry 4dl8; Hemsworth *et al.*, 2013 ▸).

A dictionary in the monomer library can accurately describe only a single conformation of a metal-containing component, for example the trigonal bipyramidal coordination of the aluminium centre in aluminium chloride, which is the default option in the library. However, the *MetalCoord* program analyses the ideal bond geometry while considering the metal environment when an input structure is provided (see Appendix *D*1). This allows the definition of a component dictionary and restraints suited to a particular chemical context, for example the octahedral coordination in the dUTPase example.

### 4.4. Ferricyanide

Due to the suboptimal treatment of metal atoms in the past, the harmonic restraints for the ferricyanide ions [Fe(CN)_6_]^3−^ (monomer code FC6, with partial occupancy) in the crystal structure of bilirubin oxidase (Koval’, Švecová *et al.*, 2019 ▸; Malý *et al.*, 2020 ▸) were applied in conjunction with a modified component dictionary to prevent geometric distortion. This type of restraint fixes atoms to their current positions, which is generally not an appropriate approach. *MetalCoord* now provides information to define more appropriate restraints and dictionaries based on ligand chemistry, which can be considered a more relevant refinement strategy. The result of the re-refinement is shown in Fig. 3 ▸(*f*).

Furthermore, in this structure, additional restraints were generated for copper cations. However, the automatic decision on their coordination number as four proved to be incorrect. To optimize the *MetalCoord* run, the option -c 3 was used to reset the maximum coordination number.

### 4.5. Zinc and haem in nitric oxide reductase

The crystal structure of nitric oxide reductase (PDB entry 3ayf; Matsumoto *et al.*, 2012 ▸) contains a zinc ion which interacts with a water molecule placed close to the iron centre of the haem molecule. *MetalCoord* analysis of the zinc ion reported two possible coordinations: trigonal-bipyramid or square-pyramid. Thus, two independent refinements were performed in *Servalcat* when using restraints based on either of these two options. Both coordination possibilities were indistinguishable in the resulting coordinates, given the data quality.

## 5. Conclusions and future perspectives

This contribution addresses one of the longstanding challenges within the *CCP*4 suite, and perhaps within the broader field of atomic structure derivation, for molecules containing metal-containing compounds. With the aid of the current *AceDRG*, *MetalCoord* and *Servalcat* software, the refinement of ligands with metals should now be semi-automatic. Given the versatility of metals and their responsiveness to environmental variations, it is recommended to generate and apply restraints specific to each structure under study before each refinement session. This approach will ensure that the correct coordination geometry is identified and utilized.

In many cases, it may be reasonable to define the component without the metal and then add the metals as separate components. If this approach is adopted, *MetalCoord* should be used in the *stats* mode. The program will then generate appropriate restraints for each metal atom based on its current environment. This approach would be effective in many cases; however, it is still neccessry to derive an accurate monomer library distributed by *CCP*4.

Although the metal-containing components in the current version of the monomer library can be considered to be satisfactory, there is much more work to be done. One future direction should involve comparative analyses of metal-coordination geometries between small-molecule databases (such as COD) and the PDB. In the current work, we used a naive, agnostic approach with the assumption that metals in macromolecules and small molecules are equally distributed. However, it is likely that biological macromolecules utilize metals that are readily available in the environment where the organism resides. To prioritize research and methodological developments aimed at improving macromolecular structures, it is necessary to conduct statistical and comparative analyses of small-molecule and macromolecular structure databases.

Another important direction is the validation of metal environments in deposited structures. While there are well established validation tools and protocols for proteins and DNA/RNA, and some exist for ligands, particularly for bonding and nonbonding interactions, such tools are not yet available for metals and their environments. The resources within *MetalCoord* could be further utilized for this purpose. Additionally, validation of charges in the local environment might also aid in the correct interpretation of chemistry. In light of the application of machine-learning techniques to derive, interpret and predict structures, chemically accurate structure derivation and annotations are more important than ever.

In the context of X-ray crystallography, further validation could be achieved through anomalous scattering, making it crucial to retain Friedel pairs in PDB submissions.

During data acquisition (using X-rays, electrons or even neutrons), metals may undergo changes in oxidation states, altering their coordination geometry (Carugo & Carugo, 2005 ▸; Yano *et al.*, 2005 ▸; Hattne *et al.*, 2018 ▸). While *MetalCoord* can generate restraints for uniform oxidation-state changes, partial oxidation presents a challenge due to the coexistence of multiple coordination geometries. To account for this, *MetalCoord* can generate restraints for user-defined alternative conformations corresponding to different oxidation states. Semi-automation of this process will require the integration of tools such as molecular graphics, chemoinformatics and precise difference-map calculations. *MetalCoord* will serve as a key component within this integrated workflow.

Another important issue that is easy to underestimate is the communication between depositors and the PDB. Enhancing this communication is essential to ensure the accuracy and reliability of metal-containing structures. While the deposition process for non-metal-containing components has seen substantial improvements, considerable work remains to optimize the deposition and documentation of metal-containing components.

## Supplementary Material

List of all coordination classes within metalCoord. DOI: 10.1107/S2059798324011458/von5002sup1.csv

Full list of frequencies of coordination classes for each metal. DOI: 10.1107/S2059798324011458/von5002sup2.csv

Idealized coordinates of coordination classes. DOI: 10.1107/S2059798324011458/von5002sup3.tgz

Supporting tables of restraints and refinement statistics. DOI: 10.1107/S2059798324011458/von5002sup4.pdf

This archive contains re-refined macromolecular structures presented in this article. The JSON files describing metal coordination were generated using MetalCoord. These JSON files were converted to restraints using the included script json2restraints.py. The structures were re-refined using Servalcat.: https://doi.org/10.5281/zenodo.13694559

## Figures and Tables

**Table 1:** The number of entries at each filtering stage

Stage	No. of entries
No. of cases from COD	429579
No. of single-metal cases	228063
No. of metal–metal bonded cases	201516
No. of used files	228063
No. of classified files	189671

**Table 2:** Most frequent coordination classes This table lists a subset of the current coordination geometry classes available within *MetalCoord*, focusing on those with at least 20 occurrences in the COD. A table with all classes is available in the supporting information. The ‘Class’ column provides the class name. The ‘Added’ column indicates whether the class was pre-existing or newly identified through analysis. The names of the added classes are relatively arbitrary. The ‘Crd’ column shows the coordination number. The ‘COD’ column lists the COD code for an example of the metal coordination. This column is empty for pre-existing classes. The ‘Used’ column indicates whether this class is currently used by *MetalCoord*. The ‘N COD’ column shows the number of occurrences of the coordination geometry in the analysed COD data. Normalized coordination geometries (with bond lengths between the metal and surrounding atoms set to 1 Å) can be found in the supporting information. Note that not all coordination geometries currently utilized by *MetalCoord* are included in this table. Those with fewer occurrences in the COD, while still employed by *MetalCoord*, are provided in the supporting information.

Class	Added	Crd	COD	Used	N COD
Bent	No	2		Yes	278
Linear	No	2		Yes	2107
Pyramid	No	3		Yes	1680
Trigonal-planar	No	3		Yes	3246
T-shape	No	3		Yes	1613
Square-non-planar	Yes	4	1508613	Yes	103
Square-planar	No	4		Yes	27649
Bicapped-linear	Yes	4	7003868	Yes	62
Trigonal-pyramid	No	4		Yes	3097
Tetrahedral	No	4		Yes	24030
Square-pyramid	No	5		Yes	15506
Tricapped-trigonal-planar	Yes	5	4070511	Yes	520
Bicapped-trigonal-planar	Yes	5	7118101	Yes	88
Trigonal-bipyramid	No	5		Yes	8945
Sandwich_4h_2	Yes	6	1558778	Yes	25
Octahedral	No	6		Yes	81057
Sandwich_4_2	Yes	6	1558752	Yes	36
Sandwich_5_1	Yes	6	4110095	Yes	63
Trigonal-prism	No	6		Yes	910
Bicapped-square-planar	Yes	6	1507592	Yes	1432
Sandwich_4h_3	Yes	7	4083238	No	160
Sandwich_4_3	Yes	7	4075391	Yes	35
Sandwich_5_2	Yes	7	7227676	Yes	595
Pentagonal-bipyramid	No	7		Yes	466
Elongated-triangular-bipyramid	Yes	8	4074228	Yes	534
Dodecahedral	No	8		Yes	923
Bicapped-octahedral	Yes	8	4069664	Yes	95
Square-antiprismatic	No	8		Yes	953
Sandwich_5h_3	Yes	8	7013773	No	51
Sandwich_6_2	Yes	8	4067378	Yes	129
Sandwich_5_3	No	8		Yes	3441
Hexagonal-bipyramid	No	8		Yes	275
Cubic	No	8		Yes	129
Sandwich_5_4h	Yes	9	4081578	No	196
Sandwich_6_3	Yes	9	7021276	Yes	1787
Sandwich_5_4	No	9		Yes	198
Sandwich_5_tricapped_i	Yes	9	4078534	No	75
Sandwich_5_tricapped_v	Yes	10	4063750	No	100
Sandwich_5_5	No	10		Yes	4852
Sandwich_7_3	Yes	10	4070696	Yes	18
Sandwich_5_square_pyramid	Yes	10		No	104
Sandwich_6_5	No	11		Yes	274
Sandwich_5_4h_v	Yes	11	4077522	No	18
Sandwich_5_5_i	Yes	11	4063984	No	160
Sandwich_8_3	Yes	11	4338644	Yes	18
Sandwich_7_5	Yes	12	4070477	Yes	54
Sandwich_6_6	No	12		No	110
Paired-octahedral	Yes	12	7005479	Yes	21
Sandwich_5_5_v	Yes	12	4067609	No	888
Sandwich_5_5_vi	Yes	13	4064274	No	184
Sandwich_8_5_i	Yes	14	4063637	No	22
Sandwich_5_5_4	Yes	14	4064647	No	99
Sandwich_8_8	Yes	16	2004620	Yes	40

**Table 3:** Most frequent coordination classes for each metal element The cases with more than 500 occurrences are present in this table. The full table can be found in the supporting information. This table may be useful for constructing a prior probability distribution for metal identification.

Metal	Class	No. of entries
Ag	Trigonal-planar	513
Ag	Tetrahedral	1052
Al	Octahedral	960
Al	Tetrahedral	2353
Au	Linear	1264
Au	Square-planar	1270
Bi	Octahedral	769
Cd	Tetrahedral	824
Cd	Octahedral	3245
Co	Square-pyramid	541
Co	Trigonal-bipyramid	861
Co	Tetrahedral	1723
Co	Octahedral	9884
Cr	Octahedral	2072
Cu	Trigonal-planar	1428
Cu	Trigonal-bipyramid	1674
Cu	Tetrahedral	2696
Cu	Square-planar	4852
Cu	Octahedral	5622
Cu	Square-pyramid	6865
Fe	Sandwich_5_3	551
Fe	Square-pyramid	605
Fe	Trigonal-bipyramid	695
Fe	Tetrahedral	1123
Fe	Sandwich_5_5	4422
Fe	Octahedral	8165
Hg	Tetrahedral	505
Ir	Sandwich_5_3	1007
Ir	Octahedral	1971
K	Elongated-triangular-bipyramid	534
Li	Tetrahedral	1382
Mg	Octahedral	1063
Mn	Square-pyramid	534
Mn	Octahedral	6789
Mo	Tetrahedral	874
Mo	Square-pyramid	1112
Mo	Octahedral	12959
Na	Octahedral	1833
Ni	Square-planar	4438
Ni	Octahedral	7294
Os	Octahedral	576
Pb	Octahedral	517
Pd	Square-planar	8287
Pt	Octahedral	785
Pt	Square-planar	6328
Re	Octahedral	2652
Rh	Octahedral	585
Rh	Sandwich_5_3	599
Rh	Bicapped-square-planar	861
Rh	Square-planar	913
Ru	Sandwich_5_3	593
Ru	Square-pyramid	733
Ru	Sandwich_6_3	1280
Ru	Octahedral	1812
Sb	Octahedral	1571
Sn	Octahedral	1025
Sn	Trigonal-bipyramid	1198
Sn	Tetrahedral	1221
Ti	Octahedral	734
V	Octahedral	836
W	Octahedral	1667
Zn	Trigonal-pyramid	525
Zn	Square-pyramid	1850
Zn	Trigonal-bipyramid	1984
Zn	Octahedral	3355
Zn	Tetrahedral	7408

## Footnotes

1 Given that multiple *R* factors may be listed in a COD entry and not all may be present, we selected crystal structures where at least one of the *R* factors is less than 0.1.

2 Here we use the term ‘user’ loosely; it can refer to an actual human user or another program, for example *CCP*4*i*2.

3 A compound is considered to be a sandwich-like structure if it contains one or two cyclopentadienyl rings bonded to a metal.

4 A structure is considered to be haem-like if it contains at least one metal atom and four five-membered rings, each consisting of four C atoms and one N atom, with the N atoms forming bonds with the metal atom.

## Appendix A Distance and angle statistics

### A1. Mode identification and distance statistics

The metal–ligand distance distributions for metals can exhibit multiple modes. To identify these modes, determine their occurrence probabilities and estimate the widths (standard deviation) of the corresponding modes, we use the following procedure.

Silverman’s method (Silverman, 1981 ▸) is used to determine the number and approximate locations of the modes. This method applies kernel density estimation to the data, resulting in a smooth empirical density. The local maxima of this density are found using simple scanning methods. Different kernel sizes are tested and the smallest bandwidth that results in the specified number of modes is selected.

Once the number and approximate positions of the modes have been identified, a Gaussian mixture model (GMM; see, for example, Bishop, 2006 ▸) method is used to optimize the mode positions, the probabilities of the occurrences of modes and their widths.

## Appendix B Symmetrized von Mises distribution

Given the circular nature of bond angles, the von Mises distribution (see, for example, Dryden & Mardia, 2016 ▸) is used to model their distribution,

where α_0_ is the mean angle, *X* is a measure of the concentration and *I*_0_ is the zeroth-order modified Bessel function of the first kind. Bond angles are generally symmetric, meaning that the function of the angle is symmetric: *f*(α) = *f*(−α) = *f*(2π − α). Therefore, a symmetrized von Mises distribution seems to be more appropriate (if the angle is around π, then all observed angles will be less than π, and therefore the estimated angle will be less than π),

It can be verified that this distribution is symmetric around 0 and π.

The symmetrized von Mises distribution can also be conveniently expressed as

where *X*_1_ = *X* cos α_0_, *X*_1_ = *X* sin α_0_ and .

With a given data set of angles the parameters *X*_1_ and *X*_2_ are estimated using maximum-likelihood estimation (MLE).

### b1. Maximum-likelihood estimation (MLE)

For the ordinary von Mises distribution, the negative log-likelihood function is

where *K* is the number of data points and α_*i*_ are the observed angles.

Expressed in terms of *X*_1_ and *X*_2_,

Define

The negative log likelihood becomes

The minimum of LL_0_ is found, and the corresponding values of *X*_1_ and *X*_2_ are used to estimate the angle and width parameters using the relationships

with *m*(*X*) = *I*_1_(*X*)/*I*_0_(*X*).

### b2. Symmetrized von Mises estimation

To estimate α_0_ near π, we use the symmetrized distribution. The minus log likelihood is

Once LL_1_ has been minimized the values of *X*_1_ and *X*_2_ are used to estimate α_0_ and *X*.

### b3. Algorithm for bond-angle estimation

For *K* observations , initial values of α_0_ and *X* are estimated using the method of moments.

(i) Compute:
(ii) Calculate (accounting for the sign appropriately):
(iii) Solve for *X*:

These initial values are further refined using the Fisher scoring method. The Newton–Raphson optimization method was found to be fast and accurate for this case.

## Appendix C Procrustes matching method

### c1. Overview of Procrustes matching

Procrustes matching is a statistical technique (Dryden & Mardia, 2016 ▸; Crosilla *et al.*, 2019 ▸) that is used to compare and align two or more shapes by eliminating differences in location, scale and orientation. This method is extensively applied in fields such as structural biology, morphometrics, computer vision and other areas where shape analysis is crucial. The primary objective of the Procrustes method is to achieve the optimal superimposition of two sets of points, ensuring that the corresponding points are as close as possible, according to the least-squares criterion.

### c2. Procrustes method formulation

Given two sets of points, **X** = [*x*_1_, *x*_2_, …, *x*_*n*_] and **Y** = [*y*_1_, *y*_2_, …, *y*_*n*_], where *x*_*i*_, *y*_*i*_ ∈ **R**^*m*^ are coordinates of points in *m*-dimensional space, the Procrustes method minimizes the objective function

where *b* is a scaling factor, **R** is an orthogonal rotation matrix, **t** is an *m*-dimensional translation vector, *∥* · *∥*_F_ denotes the Frobenius norm and .

The objective is to find the optimal parameters *b*, **R** and **t** that align configuration **X** to configuration **Y** such that the sum of squared distances between corresponding points is minimized.

### c3. Steps in Procrustes matching

(i) *Centring*. Remove translational differences by centring both configurations at the origin:
(ii) *Optimal rotation*. Compute the optimal rotation matrix **R** by solving
  where  is the singular value decomposition (SVD) of the cross-covariance matrix.
(iii) *Scale factor*. Compute the scale factor:
(iv) *Translation vector*. Compute the translation vector:
(v) *Align the configurations*. Apply the optimal rotation to align the centred and scaled configurations:

### c4. Determining optimal correspondence by permutations

When only the correspondence between metal atoms is known, and the specific correspondence between other atoms in the structures is unknown, we test all possible permutations of the points in one configuration relative to the other. The objective is to find the permutation that minimizes the Procrustes distance. It is important to note that this method is feasible for a small number of points, which is applicable in our case. The maximum number of atoms, excluding the metal, is 24, but this number is rarely more than ten. For a larger number of points, alternative methods, such as the stochastic Procrustes method, should be considered.

(i) *Generate permutations*. For a given set of points, generate all possible permutations of the points in configuration **X** with respect to the points in configuration **Y**.
(ii) *Compute the Procrustes distance for each permutation*. For each permutation **X**^σ^ of configuration **X**, compute the Procrustes distance:
(iii) *Select optimal correspondence*. Identify the permutation σ* that yields the minimum Procrustes distance:

### c5. Resulting Procrustes distance

The final Procrustes distance, with the optimal correspondence between atoms, is given by

where tr(**Σ**) is the sum of the singular values obtained from the SVD corresponding to the optimal permutation.

## Appendix D Details of extraction of metal-coordination information

### d1. Extraction of the metal environment from the component dictionary and the macromolecular model

To determine the coordination geometry around the metal in the macromolecular model, the model file (typically PDB or mmCIF) and/or the monomer CIF file containing the component of interest must first be read and processed. In *stats* mode, the monomer CIF file is not required.

In the *update* mode, if more than one instance of the metal-containing component is present in the model file, the one with the lowest *B* value and the highest average occupancy is selected. In this mode, the monomer CIF file is read first and each metal in the component is considered one by one. For each metal, the bonds to the metal and the corresponding bonded atoms are saved in a separate object. If at least one of the atoms bonded to any of the metal atoms is absent in the model file, the program terminates with an appropriate error message. Atoms from the model file that are close to the metal atoms are also added to the object. Before addition, further filtering is performed (see below).

In the *stats* mode, only the macromolecular model file is read. All instances of the metal-containing component are considered one after another. Again, each metal within the specified component is considered. All atoms in the model file, including the component itself, are added to an object if they are close to the considered metal (the neighbour list is calculated using *GEMMI*). Filtering is again applied to reduce the probability of selecting incorrect atoms.

When adding atoms to the tentative list of atoms potentially forming bonds with the metal, their alternative location must match if both the metal and the considered atom have an alt loc code.

Atoms around the metal are selected using the following filtering procedure (filtering is applied only to those atoms that are from the model file but are not in the list of bonded atoms to the metal).

(i) Set parameters: α (default = 1.5), the list β_1_ (default = [1.2, 1.3, 1.4]), α_1_ (default = 1.1), γ_1_ (default = 60°) and *N*_max_ (default = 100).
(ii) Initialize *k* (default = 3) and set β_*c*_ = β_1_[*k*].
(iii) Select all atoms for which *d*(*m*, *i*) < α(*r*_*m*_ + *r*_*i*_), where *m* denotes the metal atoms and *i* denotes all other atoms. *d*(*m*, *i*) is the calculated distance and *r*_*m*_ and *r*_*i*_ are the ‘ideal’ covalent radii of the respective atoms. Denote this set as *n*_0_.
(iv) Select all atoms for which *d*(*m*, *i*) ≤ α_1_(*r*_*m*_ + *r*_*i*_). Denote this set as *n*_1_. Add all atoms bonded to the metal, as defined in the monomer CIF file, to *n*_1_.
(v) Remove atoms in *n*_1_ from *n*_0_.
(vi) Calculate all angles: γ(*i*, *m*, *j*), where *m* is the metal and *i* and *j* are in *n*_0_ or *n*_1_. If min γ(*i*, *m*, *j*) > γ_1_ then add all remaining atoms to *n*_1_ and finish.
(vii) Find the minimum angle: γ_min_ = min γ(*i*, *m*, *j*) for all atom pairs where at least one of *i* or *j* is in *n*_0_. If γ_*min*_ < γ_1_, then take the atom with the larger coefficient, where the coefficient is *c*_*i*_ = *d*(*m*, *i*)/(*r*_*m*_ + *r*_*i*_), *c*_*j*_ = *d*(*m*, *j*)/(*r*_*m*_ + *r*_*j*_). If this atom is in *n*_0_ and max(*c_i_*, *c_j_*) > β*_c_*, then remove this atom from *n*_0_.
(viii) Repeat step (vii) until there are no more changes.
(ix) Decrease *k* by 1 and set β_*c*_ = β_1_[*k*].
(x) Repeat steps (vii)–(ix) until there are no more changes.
(xi) Add all remaining atoms in *n*_0_ to *n*_1_.
(xii) Keep only the *N*_max_ neighbours with the smallest coefficients.If *n*_1_ has fewer than *N*_max_ atoms, do not remove anything.

If the symmetry-related atoms are making contact with the metal then the following additional procedure is used [note that all atoms are considered in step (i) and then the remaining atoms are considered in step (ii)].

(i) If *q*_*m*_ + *q*_*i*_ > 1 then this atom is added to the list. Here, *q*_*m*_ is the occupancy of the metal and *q*_*i*_ is the occupancy of the considered symmetry-related atom. Atom *j* of the same asymmetric unit has already been added to the list.
(ii) If *q*_*i*_ + *q*_*j*_ > *q*_*m*_ and *i* and *j* are the same but symmetry-related atoms then do not add this symmetry-related atom. Again the atom *i* in the same asymmetric unit as the metal atom has already been added to the list.

Here, we define occupancy as the crystallographic occupancy: the proportion of the atom in the asymmetric unit.

### d2. Matching the metal environment with the coordination library

To accurately characterize the metal environments, we employ a combinatorial Procrustes matching procedure that aligns the metal and its environment (derived as described above) with idealized coordinates from the coordination library. In the current implementation, we use only 54 out of the 95 coordination classes. Currently, we exclude coordination classes where nonmetal atoms are bonded to each other. The exceptions are ‘sandwich-like structures’, where all atoms of at least one ring form bonds with the metal.

### d3. Compiling statistics: bonds

The program uses several strategies for compiling bond and angle statistics. For calculations of bond statistics, the following strategies are used.

(i) *Exact structural match*. Identifies a perfect match between two structures in terms of geometric arrangement, element types, atom counts and spatial positions. This is the strictest criterion and establishes a direct one-to-one correspondence between all atoms of the metal–ligand complexes.
(ii) *Exact element match*. Matches two structures based on having the same geometric arrangement, element types and atom counts, regardless of exact spatial positions. This method allows for minor spatial deviations while maintaining a strict correspondence of elements.
(iii) *Partial element match*. Matches structures that belong to the same geometric class and share the same types of elements, even if the element counts differ. Allows for flexibility in both element counts and spatial arrangements, accommodating more diverse coordination environments.
(iv) *Geometric class match*. Matches structures based on their geometric class (for example tetrahedral, octahedral) and requires at least one common element. Focuses on maintaining the overall shape and coordination geometry rather than matching specific atoms or counts.
(v) *Coordination number match*. Ensures that the structures being compared have the same coordination number (*i.e.* the number of atoms directly bonded to the metal centre) and at least one shared element. Prioritizes the preservation of coordination geometry.
(vi) *Global distance statistics*. A fallback strategy that calculates average distance statistics from all available metal-atom pairs in the data set when no specific structural match can be found. Provides a general benchmark for metal–ligand distances across various environments.
(vii) *Covalent radii sum*. A last resort if none of the above strategies works. In this case, the sum of the covalent radii of the metal and ligand atoms is used to estimate bond distances.

### d4. Compiling statistics: angles

For calculations of angle statistics, the following strategies are used.

(i) *Exact structural match*. For structures with an exact structural match, angles are calculated based on the matched geometries. This ensures the highest accuracy in representing the angles in the coordination environment.
(ii) *Angles from idealized coordinates*. If an exact structural match is not available, angles are derived from the idealized coordination geometries. This approach uses theoretical models to estimate the angles.
(iii) *Default angles*. When no coordination match is found, angle information is not provided.
