# Supplementary material for: Improving macromolecular structure refinement with metal-coordination restraints
Source: Acta Crystallogr D Struct Biol. 2024 Dec 3;80(Pt 12):821–33. doi: 10.1107/S2059798324011458 (PMC11626771; doi:10.1107/S2059798324011458)
Supplement: Supplementary file 4 [file d-80-00821-sup4.pdf]

STRUCTURAL BIOLOGY

Volume 80 (2024)

Supporting information for article:

Improving macromolecular structure refinement with metal-coordination restraints

Kaveh H. Babai, Fei Long, Martin Malý, Keitaro Yamashita and Garib N. Murshudov

Table S1 Refinement statistics of the example structures. CC*F* denotes the number-weighted average correlation coefficient, within resolution bins, between observed and calculated structure factor amplitudes.

6wj6

Resolution (Å)	2.58
FSCaverage	0.84

1w9m

Resolution range (Å)	59.88 – 1.35	1.37 – 1.35
*R*work / *R*free	0.1381 / 0.1640	0.1908 / 0.2173
CC*F*work / CC*F*free	0.9433 / 0.9204	0.8980 / 0.8498

2ynm

Resolution range (Å)	34.51 – 2.10	2.15 – 2.10
*R*work / *R*free	0.2052 / 0.2507	0.2777 / 0.3343
CC*F*work / CC*F*free	0.8346 / 0.7539	0.7307 / 0.5385

4dl8

Resolution range (Å)	28.20 – 1.70	1.78 – 1.70
*R*work / *R*free	0.1603 / 0.1842	0.2223 / 0.2358
CC*F*work / CC*F*free	0.9225 / 0.8972	0.8725 / 0.8400

6i3j

Resolution range (Å)	113.49 – 2.59	2.70 – 2.59
*R*work / *R*free	0.1620 / 0.2026	0.2627 / 0.3182
CC*F*work / CC*F*free	0.9118 / 0.8479	0.8069 / 0.6641

Table S2 Metal sites shown in the examples in Fig. 3 and corresponding restraints used for structure refinement.

6wj6

**Metal atom**	**Geometry class**	**No. coordination**	**Procrustes distance**	**No. reference structures**
D/CLA 402/MG	square-pyramid	5	0.171	112
E/HEM 101/FE	octahedral	6	0.248	96,000

Ideal distances:

**Metal atom**	**Atom**	**Ideal distance(s) (Å)**	**SD (Å)**
D/CLA 402/MG	D/CLA 402/NB	2.09	0.04
D/CLA 402/MG	D/CLA 402/NC	2.09	0.04
D/CLA 402/MG	D/CLA 402/ND	2.09	0.04
D/CLA 402/MG	D/CLA 402/NA	2.09	0.04
D/CLA 402/MG	A/HOH 523/O	2.04, 2.07	0.02, 0.02
E/HEM 101/FE	E/HEM 101/NC	1.97, 2.17	0.04, 0.05
E/HEM 101/FE	E/HEM 101/ND	1.97, 2.17	0.04, 0.05
E/HEM 101/FE	E/HEM 101/NA	1.97, 2.17	0.04, 0.05
E/HEM 101/FE	E/HEM 101/NB	1.97, 2.17	0.04, 0.05
E/HEM 101/FE	E/HIS 23/NE2	1.97, 2.17	0.04, 0.05
E/HEM 101/FE	F/HIS 23/NE2	1.97, 2.17	0.04, 0.05

Ideal angles:

**Atom 1**	**Metal atom**	**Atom 3**	**Ideal angle (°)**	**SD (°)**
D/CLA 402/NB	D/CLA 402/MG	D/CLA 402/NC	87.68	4.56
D/CLA 402/NB	D/CLA 402/MG	D/CLA 402/ND	156.75	5.59
D/CLA 402/NB	D/CLA 402/MG	D/CLA 402/NA	87.68	4.56
D/CLA 402/NB	D/CLA 402/MG	A/HOH 523/O	101.41	4.16
D/CLA 402/NC	D/CLA 402/MG	D/CLA 402/ND	87.68	4.56
D/CLA 402/NC	D/CLA 402/MG	D/CLA 402/NA	156.75	5.59
D/CLA 402/NC	D/CLA 402/MG	A/HOH 523/O	101.41	4.16
D/CLA 402/ND	D/CLA 402/MG	D/CLA 402/NA	87.68	4.56
D/CLA 402/ND	D/CLA 402/MG	A/HOH 523/O	101.41	4.16
D/CLA 402/NA	D/CLA 402/MG	A/HOH 523/O	101.41	4.16
E/HEM 101/NC	E/HEM 101/FE	E/HEM 101/ND	90.10	6.05
E/HEM 101/NC	E/HEM 101/FE	E/HEM 101/NA	180.00	9.32
E/HEM 101/NC	E/HEM 101/FE	E/HEM 101/NB	90.10	6.05
E/HEM 101/NC	E/HEM 101/FE	E/HIS 23/NE2	90.10	6.05
E/HEM 101/NC	E/HEM 101/FE	F/HIS 23/NE2	90.10	6.05
E/HEM 101/ND	E/HEM 101/FE	E/HEM 101/NA	90.10	6.05
E/HEM 101/ND	E/HEM 101/FE	E/HEM 101/NB	180.00	9.32
E/HEM 101/ND	E/HEM 101/FE	E/HIS 23/NE2	90.10	6.05
E/HEM 101/ND	E/HEM 101/FE	F/HIS 23/NE2	90.10	6.05
E/HEM 101/NA	E/HEM 101/FE	E/HEM 101/NB	90.10	6.05
E/HEM 101/NA	E/HEM 101/FE	E/HIS 23/NE2	90.10	6.05
E/HEM 101/NA	E/HEM 101/FE	F/HIS 23/NE2	90.10	6.05
E/HEM 101/NB	E/HEM 101/FE	E/HIS 23/NE2	90.10	6.05
E/HEM 101/NB	E/HEM 101/FE	F/HIS 23/NE2	90.10	6.05
E/HIS 23/NE2	E/HEM 101/FE	F/HIS 23/NE2	173.78	6.99

1w9m

Metal atom	Geometry class	No. coordination	Procrustes distance	No. reference structures
A/FS2 1555/FE5	tetrahedral	4	0.090	464
A/FS2 1555/FE6	tetrahedral	4	0.131	464
A/FS2 1555/FE7	trigonal-bipyramid	5	0.076	576
A/FS2 1555/FE8	trigonal-bipyramid	5	0.157	426

Ideal distances:

Metal atom	Atom	Ideal distance(s) (Å)	SD (Å)
A/FS2 1555/FE5	A/FS2 1555/S5	2.33	0.06
A/FS2 1555/FE5	A/FS2 1555/O9	1.90	0.1
A/FS2 1555/FE5	A/FS2 1555/S6	2.33	0.06
A/FS2 1555/FE5	A/CYS 434/SG	2.33	0.06
A/FS2 1555/FE6	A/FS2 1555/S5	2.33	0.06
A/FS2 1555/FE6	A/FS2 1555/O8	1.90	0.06
A/FS2 1555/FE6	A/FS2 1555/S6	2.33	0.06
A/FS2 1555/FE6	A/CYS 312/SG	2.33	0.06
A/FS2 1555/FE7	A/FS2 1555/O9	1.99	0.13
A/FS2 1555/FE7	A/FS2 1555/O1	1.99	0.13
A/FS2 1555/FE7	A/HIS 244/NE2	2.12	0.13
A/FS2 1555/FE7	A/GLU 268/OE2	1.99	0.13
A/FS2 1555/FE7	A/CYS 459/SG	2.30	0.09
A/FS2 1555/FE8	A/FS2 1555/O1	1.99	0.13
A/FS2 1555/FE8	A/FS2 1555/S6	2.30	0.09
A/FS2 1555/FE8	A/FS2 1555/O8	1.99	0.13
A/FS2 1555/FE8	A/CSS 406/SD	2.30	0.09
A/FS2 1555/FE8	A/GLU 494/OE2	1.99	0.13

Ideal angles:

**Atom 1**	**Metal atom**	**Atom 3**	**Ideal angle (°)**	**SD (°)**
A/FS2 1555/S5	A/FS2 1555/FE5	A/FS2 1555/O9	109.47	5.00
A/FS2 1555/S5	A/FS2 1555/FE5	A/FS2 1555/S6	109.47	5.00
A/FS2 1555/S5	A/FS2 1555/FE5	A/CYS 434/SG	109.47	5.00
A/FS2 1555/O9	A/FS2 1555/FE5	A/FS2 1555/S6	109.47	5.00
A/FS2 1555/O9	A/FS2 1555/FE5	A/CYS 434/SG	109.47	5.00
A/FS2 1555/S6	A/FS2 1555/FE5	A/CYS 434/SG	109.47	5.00
A/FS2 1555/S5	A/FS2 1555/FE6	A/FS2 1555/O8	109.47	5.00
A/FS2 1555/S5	A/FS2 1555/FE6	A/FS2 1555/S6	109.47	5.00
A/FS2 1555/S5	A/FS2 1555/FE6	A/CYS 312/SG	109.47	5.00
A/FS2 1555/O8	A/FS2 1555/FE6	A/FS2 1555/S6	109.47	5.00
A/FS2 1555/O8	A/FS2 1555/FE6	A/CYS 312/SG	109.47	5.00
A/FS2 1555/S6	A/FS2 1555/FE6	A/CYS 312/SG	109.47	5.00
A/FS2 1555/O9	A/FS2 1555/FE7	A/FS2 1555/O1	90.00	5.00
A/FS2 1555/O9	A/FS2 1555/FE7	A/HIS 244/NE2	120.00	5.00
A/FS2 1555/O9	A/FS2 1555/FE7	A/GLU 268/OE2	90.00	5.00
A/FS2 1555/O9	A/FS2 1555/FE7	A/CYS 459/SG	120.00	5.00
A/FS2 1555/O1	A/FS2 1555/FE7	A/HIS 244/NE2	90.00	5.00
A/FS2 1555/O1	A/FS2 1555/FE7	A/GLU 268/OE2	180.00	5.00
A/FS2 1555/O1	A/FS2 1555/FE7	A/CYS 459/SG	90.00	5.00
A/HIS 244/NE2	A/FS2 1555/FE7	A/GLU 268/OE2	90.00	5.00
A/HIS 244/NE2	A/FS2 1555/FE7	A/CYS 459/SG	120.00	5.00
A/GLU 268/OE2	A/FS2 1555/FE7	A/CYS 459/SG	90.00	5.00
A/FS2 1555/O1	A/FS2 1555/FE8	A/FS2 1555/S6	120.00	5.00
A/FS2 1555/O1	A/FS2 1555/FE8	A/FS2 1555/O8	90.00	5.00
A/FS2 1555/O1	A/FS2 1555/FE8	A/CSS 406/SD	90.00	5.00
A/FS2 1555/O1	A/FS2 1555/FE8	A/GLU 494/OE2	120.00	5.00
A/FS2 1555/S6	A/FS2 1555/FE8	A/FS2 1555/O8	90.00	5.00
A/FS2 1555/S6	A/FS2 1555/FE8	A/CSS 406/SD	90.00	5.00
A/FS2 1555/S6	A/FS2 1555/FE8	A/GLU 494/OE2	120.00	5.00
A/FS2 1555/O8	A/FS2 1555/FE8	A/CSS 406/SD	180.00	5.00
A/FS2 1555/O8	A/FS2 1555/FE8	A/GLU 494/OE2	90.00	5.00
A/CSS 406/SD	A/FS2 1555/FE8	A/GLU 494/OE2	90.00	5.00

2ynm

Metal atom	Geometry class	No. coordination	Procrustes distance	No. reference structures
A/AF3 1299/AL	trigonal-bipyramid	5	0.145	273
A/MG 1301/MG	octahedral	6	0.067	1,015

Ideal distances:

Metal atom	Atom	Ideal distance (Å)	SD (Å)
A/AF3 1299/AL	A/AF3 1299/F1	1.90	0.18
A/AF3 1299/AL	A/AF3 1299/F2	1.90	0.18
A/AF3 1299/AL	A/AF3 1299/F3	1.90	0.18
A/AF3 1299/AL	A/HOH 2019/O	1.83	0.09
A/AF3 1299/AL	A/ADP 1300/O2B	1.83	0.09
A/MG 1301/MG	A/AF3 1299/F1	2.06	0.12
A/MG 1301/MG	A/ADP 1300/O3B	2.07	0.04
A/MG 1301/MG	A/HOH 2009/O	2.07	0.04
A/MG 1301/MG	A/HOH 2008/O	2.07	0.04
A/MG 1301/MG	A/HOH 2010/O	2.07	0.04
A/MG 1301/MG	A/SER 43/OG	2.07	0.04

Ideal angles:

Atom 1	Metal atom	Atom 3	Ideal angle (°)	SD (°)
A/AF3 1299/F1	A/AF3 1299/AL	A/AF3 1299/F2	120.00	5.00
A/AF3 1299/F1	A/AF3 1299/AL	A/AF3 1299/F3	120.00	5.00
A/AF3 1299/F1	A/AF3 1299/AL	A/HOH 2019/O	90.00	5.00
A/AF3 1299/F1	A/AF3 1299/AL	A/ADP 1300/O2B	90.00	5.00
A/AF3 1299/F2	A/AF3 1299/AL	A/AF3 1299/F3	120.00	5.00
A/AF3 1299/F2	A/AF3 1299/AL	A/HOH 2019/O	90.00	5.00
A/AF3 1299/F2	A/AF3 1299/AL	A/ADP 1300/O2B	90.00	5.00
A/AF3 1299/F3	A/AF3 1299/AL	A/HOH 2019/O	90.00	5.00
A/AF3 1299/F3	A/AF3 1299/AL	A/ADP 1300/O2B	90.00	5.00
A/HOH 2019/O	A/AF3 1299/AL	A/ADP 1300/O2B	180.00	5.00
A/AF3 1299/F1	A/MG 1301/MG	A/ADP 1300/O3B	90.00	5.00
A/AF3 1299/F1	A/MG 1301/MG	A/HOH 2009/O	90.00	5.00
A/AF3 1299/F1	A/MG 1301/MG	A/HOH 2008/O	90.00	5.00
A/AF3 1299/F1	A/MG 1301/MG	A/HOH 2010/O	90.00	5.00
A/AF3 1299/F1	A/MG 1301/MG	A/SER 43/OG	180.00	5.00
A/ADP 1300/O3B	A/MG 1301/MG	A/HOH 2009/O	180.00	5.00

A/ADP 1300/O3B	A/MG 1301/MG	A/HOH 2008/O	90.00	5.00
A/ADP 1300/O3B	A/MG 1301/MG	A/HOH 2010/O	90.00	5.00
A/ADP 1300/O3B	A/MG 1301/MG	A/SER 43/OG	90.00	5.00
A/HOH 2009/O	A/MG 1301/MG	A/HOH 2008/O	90.00	5.00
A/HOH 2009/O	A/MG 1301/MG	A/HOH 2010/O	90.00	5.00
A/HOH 2009/O	A/MG 1301/MG	A/SER 43/OG	90.00	5.00
A/HOH 2008/O	A/MG 1301/MG	A/HOH 2010/O	180.00	5.00
A/HOH 2008/O	A/MG 1301/MG	A/SER 43/OG	90.00	5.00
A/HOH 2010/O	A/MG 1301/MG	A/SER 43/OG	90.00	5.00

4dl8

Metal atom	Geometry class	No. coordination	Procrustes distance	No. reference structures
A/AF3 302/AL	octahedral	6	0.067	33
A/MG 304/MG	octahedral	6	0.074	1,015
A/MG 305/MG	octahedral	6	0.109	1,015
A/NA 306/NA	octahedral	6	0.239	72,624

Ideal distances:

Metal atom	Atom	Ideal distance (Å)	SD (Å)
A/AF3 302/AL	A/AF3 302/F2	1.82	0.05
A/AF3 302/AL	A/AF3 302/F1	1.82	0.05
A/AF3 302/AL	A/AF3 302/F3	1.82	0.05
A/AF3 302/AL	A/PO4 303/O1	1.88	0.04
A/AF3 302/AL	A/HOH 401/O	1.88	0.04
A/AF3 302/AL	A/UMP 301/OP1	1.88	0.04
A/MG 304/MG	A/GLU 48/OE2	2.07	0.04
A/MG 304/MG	A/GLU 51/OE2	2.07	0.04
A/MG 304/MG	A/UMP 301/OP2	2.07	0.04
A/MG 304/MG	A/AF3 302/F3	2.06	0.12
A/MG 304/MG	A/HOH 437/O	2.07	0.04
A/MG 304/MG	A/PO4 303/O2	2.07	0.04
A/MG 305/MG	A/ASP 79/OD2	2.07	0.04
A/MG 305/MG	A/GLU 48/OE1	2.07	0.04
A/MG 305/MG	A/GLU 51/OE1	2.07	0.04
A/MG 305/MG	A/GLU 76/OE1	2.07	0.04
A/MG 305/MG	A/HOH 401/O	2.07	0.04
A/MG 305/MG	A/AF3 302/F3	2.06	0.12
A/NA 306/NA	A/GLU 76/OE2	2.42	0.09
A/NA 306/NA	A/HOH 402/O	2.42	0.09
A/NA 306/NA	A/HOH 403/O	2.42	0.09
A/NA 306/NA	A/GLU 51/OE1	2.42	0.09
A/NA 306/NA	A/HOH 401/O	2.42	0.09
A/NA 306/NA	A/HOH 485/O	2.42	0.09

Ideal angles:

Atom 1	Metal atom	Atom 3	Ideal angle (°)	SD (°)
A/AF3 302/F2	A/AF3 302/AL	A/AF3 302/F1	90.00	5.00
A/AF3 302/F2	A/AF3 302/AL	A/AF3 302/F3	180.00	5.00
A/AF3 302/F2	A/AF3 302/AL	A/PO4 303/O1	90.00	5.00
A/AF3 302/F2	A/AF3 302/AL	A/HOH 401/O	90.00	5.00
A/AF3 302/F2	A/AF3 302/AL	A/UMP 301/OP1	90.00	5.00
A/AF3 302/F1	A/AF3 302/AL	A/AF3 302/F3	90.00	5.00
A/AF3 302/F1	A/AF3 302/AL	A/PO4 303/O1	180.00	5.00
A/AF3 302/F1	A/AF3 302/AL	A/HOH 401/O	90.00	5.00
A/AF3 302/F1	A/AF3 302/AL	A/UMP 301/OP1	90.00	5.00
A/AF3 302/F3	A/AF3 302/AL	A/PO4 303/O1	90.00	5.00
A/AF3 302/F3	A/AF3 302/AL	A/HOH 401/O	90.00	5.00
A/AF3 302/F3	A/AF3 302/AL	A/UMP 301/OP1	90.00	5.00
A/PO4 303/O1	A/AF3 302/AL	A/HOH 401/O	90.00	5.00
A/PO4 303/O1	A/AF3 302/AL	A/UMP 301/OP1	90.00	5.00
A/HOH 401/O	A/AF3 302/AL	A/UMP 301/OP1	180.00	5.00
A/GLU 48/OE2	A/MG 304/MG	A/GLU 51/OE2	90.00	5.00
A/GLU 48/OE2	A/MG 304/MG	A/UMP 301/OP2	90.00	5.00
A/GLU 48/OE2	A/MG 304/MG	A/AF3 302/F3	90.00	5.00
A/GLU 48/OE2	A/MG 304/MG	A/HOH 437/O	90.00	5.00
A/GLU 48/OE2	A/MG 304/MG	A/PO4 303/O2	180.00	5.00
A/GLU 51/OE2	A/MG 304/MG	A/UMP 301/OP2	180.00	5.00
A/GLU 51/OE2	A/MG 304/MG	A/AF3 302/F3	90.00	5.00
A/GLU 51/OE2	A/MG 304/MG	A/HOH 437/O	90.00	5.00
A/GLU 51/OE2	A/MG 304/MG	A/PO4 303/O2	90.00	5.00
A/UMP 301/OP2	A/MG 304/MG	A/AF3 302/F3	90.00	5.00
A/UMP 301/OP2	A/MG 304/MG	A/HOH 437/O	90.00	5.00
A/UMP 301/OP2	A/MG 304/MG	A/PO4 303/O2	90.00	5.00
A/AF3 302/F3	A/MG 304/MG	A/HOH 437/O	180.00	5.00
A/AF3 302/F3	A/MG 304/MG	A/PO4 303/O2	90.00	5.00
A/HOH 437/O	A/MG 304/MG	A/PO4 303/O2	90.00	5.00
A/ASP 79/OD2	A/MG 305/MG	A/GLU 48/OE1	90.00	5.00
A/ASP 79/OD2	A/MG 305/MG	A/GLU 51/OE1	180.00	5.00
A/ASP 79/OD2	A/MG 305/MG	A/GLU 76/OE1	90.00	5.00
A/ASP 79/OD2	A/MG 305/MG	A/HOH 401/O	90.00	5.00
A/ASP 79/OD2	A/MG 305/MG	A/AF3 302/F3	90.00	5.00
A/GLU 48/OE1	A/MG 305/MG	A/GLU 51/OE1	90.00	5.00

A/GLU 48/OE1	A/MG 305/MG	A/GLU 76/OE1	90.00	5.00
A/GLU 48/OE1	A/MG 305/MG	A/HOH 401/O	180.00	5.00
A/GLU 48/OE1	A/MG 305/MG	A/AF3 302/F3	90.00	5.00
A/GLU 51/OE1	A/MG 305/MG	A/GLU 76/OE1	90.00	5.00
A/GLU 51/OE1	A/MG 305/MG	A/HOH 401/O	90.00	5.00
A/GLU 51/OE1	A/MG 305/MG	A/AF3 302/F3	90.00	5.00
A/GLU 76/OE1	A/MG 305/MG	A/HOH 401/O	90.00	5.00
A/GLU 76/OE1	A/MG 305/MG	A/AF3 302/F3	180.00	5.00
A/HOH 401/O	A/MG 305/MG	A/AF3 302/F3	90.00	5.00
A/GLU 76/OE2	A/NA 306/NA	A/HOH 402/O	90.05	8.59
A/GLU 76/OE2	A/NA 306/NA	A/HOH 403/O	90.05	8.59
A/GLU 76/OE2	A/NA 306/NA	A/GLU 51/OE1	90.05	8.59
A/GLU 76/OE2	A/NA 306/NA	A/HOH 401/O	90.05	8.59
A/GLU 76/OE2	A/NA 306/NA	A/HOH 485/O	170.75	9.36
A/HOH 402/O	A/NA 306/NA	A/HOH 403/O	90.05	8.59
A/HOH 402/O	A/NA 306/NA	A/GLU 51/OE1	90.05	8.59
A/HOH 402/O	A/NA 306/NA	A/HOH 401/O	170.75	9.36
A/HOH 402/O	A/NA 306/NA	A/HOH 485/O	90.05	8.59
A/HOH 403/O	A/NA 306/NA	A/GLU 51/OE1	170.75	9.36
A/HOH 403/O	A/NA 306/NA	A/HOH 401/O	90.05	8.59
A/HOH 403/O	A/NA 306/NA	A/HOH 485/O	90.05	8.59
A/GLU 51/OE1	A/NA 306/NA	A/HOH 401/O	90.05	8.59
A/GLU 51/OE1	A/NA 306/NA	A/HOH 485/O	90.05	8.59
A/HOH 401/O	A/NA 306/NA	A/HOH 485/O	90.05	8.59

6i3j

Metal atom	Geometry class	No. coordination	Procrustes distance	No. reference structures
A/FE2 615/FC6	octahedral	6	0.022	17,376

Ideal distances:

Metal atom	Atom	Ideal distance (Å)	SD (Å)
A/FE2 615/FC6	A/C11 615/FC6	1.93	0.03
A/FE2 615/FC6	A/C22 615/FC6	1.93	0.03
A/FE2 615/FC6	A/C23 615/FC6	1.93	0.03
A/FE2 615/FC6	A/C21 615/FC6	1.93	0.03
A/FE2 615/FC6	A/C26 615/FC6	1.93	0.03
A/FE2 615/FC6	A/C24 615/FC6	1.93	0.03

Ideal angles:

Atom 1	Metal atom	Atom 3	Ideal angle (°)	SD (°)
A/C11 615/FC6	A/FE2 615/FC6	A/C22 615/FC6	90.00	2.22
A/C11 615/FC6	A/FE2 615/FC6	A/C23 615/FC6	90.00	2.22
A/C11 615/FC6	A/FE2 615/FC6	A/C21 615/FC6	90.00	2.22
A/C11 615/FC6	A/FE2 615/FC6	A/C26 615/FC6	90.00	2.22
A/C11 615/FC6	A/FE2 615/FC6	A/C24 615/FC6	180.00	2.72
A/C22 615/FC6	A/FE2 615/FC6	A/C23 615/FC6	90.00	2.22
A/C22 615/FC6	A/FE2 615/FC6	A/C21 615/FC6	90.00	2.22
A/C22 615/FC6	A/FE2 615/FC6	A/C26 615/FC6	180.00	2.72
A/C22 615/FC6	A/FE2 615/FC6	A/C24 615/FC6	90.00	2.22
A/C23 615/FC6	A/FE2 615/FC6	A/C21 615/FC6	180.00	2.72
A/C23 615/FC6	A/FE2 615/FC6	A/C26 615/FC6	90.00	2.22
A/C23 615/FC6	A/FE2 615/FC6	A/C24 615/FC6	90.00	2.22
A/C21 615/FC6	A/FE2 615/FC6	A/C26 615/FC6	90.00	2.22
A/C21 615/FC6	A/FE2 615/FC6	A/C24 615/FC6	90.00	2.22
A/C26 615/FC6	A/FE2 615/FC6	A/C24 615/FC6	90.00	2.22
